# DEGRO practical guidelines: radiotherapy of breast cancer I: Radiotherapy following breast conserving therapy for invasive breast cancer

**DOI:** 10.1007/s00066-013-0437-8

**Published:** 2013-09-05

**Authors:** F. Sedlmayer, M.-L. Sautter-Bihl, W. Budach, J. Dunst, G. Fastner, P. Feyer, R. Fietkau, W. Haase, W. Harms, R. Souchon, F. Wenz, R. Sauer, Breast Cancer Expert Panel of the German Society of Radiation Oncology (DEGRO)

**Affiliations:** 1 Department of Radiotherapy and Radiation Oncology, LKH Salzburg, Paracelsus Medical University Hospital, Muellner Haupstr. 48, Salzburg, Austria; 2 Klinik für Radioonkologie und Strahlentherapie, Städtisches Klinikum Karlsruhe, Karlsruhe, Germany; 3 University Hospital Duesseldorf, Duesseldorf, Germany; 4 University Hospital Schleswig-Holstein, Luebeck, Germany; 5 Klinikum Neukoelln, Berlin, Germany; 6 University Hospital Erlangen, Erlangen, Germany; 7 formerly St.-Vincentius-Kliniken, Karlsruhe, Germany; 8 St. Clara Hospital, Basel, Switzerland; 9 University Hospital Tübingen, Tübingen, Germany; 10 University Hospital Mannheim, Mannheim, Germany; 11 University Hospital Erlangen, Erlangen, Germany

**Keywords:** Breast conserving therapy, Whole breast irradiation, Partial breast radiotherapy, Boost radiotherapy, Fractionation, Brusterhaltende Therapie, Ganzbrustbestrahlung, Partialbrustbestrahlung, Boostbestrahlung, Fraktionierung

## Abstract

**Background and purpose:**

The aim of the present paper is to update the practical guidelines for postoperative adjuvant radiotherapy of breast cancer published in 2007 by the breast cancer expert panel of the German Society for Radiooncology (Deutsche Gesellschaft für Radioonkologie, DEGRO). The present recommendations are based on a revision of the German interdisciplinary S-3 guidelines published in July 2012.

**Methods:**

A comprehensive survey of the literature concerning radiotherapy following breast conserving therapy (BCT) was performed using the search terms “breast cancer”, “radiotherapy”, and “breast conserving therapy”. Data from lately published meta-analyses, recent randomized trials, and guidelines of international breast cancer societies, yielding new aspects compared to 2007, provided the basis for defining recommendations according to the criteria of evidence-based medicine. In addition to the more general statements of the DKG (Deutsche Krebsgesellschaft), this paper addresses indications, target definition, dosage, and technique of radiotherapy of the breast after conservative surgery for invasive breast cancer.

**Results:**

Among numerous reports on the effect of radiotherapy during BCT published since the last recommendations, the recent EBCTCG report builds the largest meta-analysis so far available. In a 15 year follow-up on 10,801 patients, whole breast irradiation (WBI) halves the average annual rate of disease recurrence (RR 0.52, 0.48–0.56) and reduces the annual breast cancer death rate by about one sixth (RR 0.82, 0.75–0.90), with a similar proportional, but different absolute benefit in prognostic subgroups (EBCTCG 2011).

Furthermore, there is growing evidence that risk-adapted dose augmentation strategies to the tumor bed as well as the implementation of high precision RT techniques (e.g., intraoperative radiotherapy) contribute substantially to a further reduction of local relapse rates. A main focus of ongoing research lies in partial breast irradiation strategies as well as WBI hypofractionation schedules. The potential of both in replacing normofractionated WBI has not yet been finally clarified.

**Conclusion:**

After breast conserving surgery, no subgroup even in low risk patients has yet been identified for whom radiotherapy can be safely omitted without compromising local control and, hence, cancer-specific survival. In most patients, this translates into an overall survival benefit.

## Abstract (de)

**Zielsetzung:**

Aktualisierung der DEGRO-Leitlinien von 2007 zur Radiotherapie (RT) invasiver Mammakarzinome nach brusterhaltender Operation. Die hier präsentierten Empfehlungen basieren auf der interdisziplinären S3-Leitlinie der Deutschen Krebsgesellschaft, deren aktuelle Version 2012 publiziert wurde.

**Methodik:**

Die DEGRO-Expertengruppe Mammakarzinom führte eine Literaturrecherche aktueller klinisch kontrollierter Studien, systematischer Reviews und Metaanalysen sowie Leitlinien internationaler Gesellschaften im Hinblick auf neue Aspekte gegenüber 2007 durch, wobei sie sich an den Kriterien evidenzbasierter Medizin orientierte. Suchbegriffe waren „breast cancer“, „radiotherapy“ und „breast conserving therapy“. Ergänzend zu den allgemeineren Statements der S3-Leitlinie zielt diese Arbeit auf Indikationsstellung, Zielvolumendefinition, Dosierung und Techniken einer Bestrahlung nach brusterhaltender Operation invasiver Mammakarzinome.

**Ergebnisse:**

Von den zahlreichen Publikationen, die im Intervall seit den letzten DEGRO-Empfehlungen erschienen sind, stellt der rezente EBCTCG-Bericht aus dem Jahr 2011 die bislang zahlenmäßig größte Metaanalyse dar. In einer 15-Jahres-Nachbeobachtung an 10.801 Patienten konnte gezeigt werden, dass eine Ganzbrustbestrahlung die jährliche Rezidivrate halbiert (RR 0,52; 0,48–0,56) und die jährliche Rate der Brustkrebssterblichkeit um ein Sechstel reduziert (RR 0,82; 0,75–0,90), mit ähnlicher proportionaler, aber unterschiedlicher absoluter Ausprägung in prognostischen Untergruppen (EBCTCG 2011).

Darüber hinaus besteht zunehmende Evidenz, dass risikoadaptierte Strategien einer Dosiserhöhung im Tumorbett sowie die Anwendung hochpräziser RT-Techniken (z. B. IORT) entscheidend zu einer weiteren Reduktion der Lokalrezidivraten beitragen. Ein Schwerpunkt der aktuellen Forschung liegt in der Evaluierung von Techniken der Partialbrustbestrahlung sowie im Einsatz hypofraktionierter Dosierungsschemata in der Ganzbrustbestrahlung. Das Potenzial beider Strategien, eine normofraktionierte Ganzbrustbestrahlung zu ersetzen, ist derzeit nicht abschließend geklärt.

**Schlussfolgerungen:**

Nach brusterhaltender Operation invasiver Mammakarzinome konnte selbst bei Niedrigrisiko-Patientinnen bislang keine Subgruppe identifiziert werden, bei der auf eine nachfolgende Radiotherapie verzichtetet werden kann, ohne die lokale Tumorkontrolle und in weiterer Folge das krebsspezifische Überleben zu beeinträchtigen. Überdies resultiert bei den meisten Patientinnen durch die Bestrahlung ein Überlebensvorteil.

The evidence for the benefits of postoperative radiotherapy to the whole breast (WBI) has been further substantiated since the last recommendations on the basis of updated meta-analyses, systematic reviews, and randomized controlled trials. Depending on tumor stage, WBI reduces in-breast recurrences as well as regional relapses [23, 24, 27]. To date, there are no conclusive data from prospective clinical investigations about predictive genetic and/or molecular markers on response to radiotherapy

Endocrine therapy can reduce or delay locoregional recurrences, however, not distinctive and sustainable enough to compensate the deterioration of local control when WBI is omitted in any age or risk group [27, 68, 77].

 

**Statement RT 1** (Leitlinienprogramm 2012) [74]

In invasive breast carcinoma, postoperative radiotherapy following breast conserving surgery has to be performed (LoE 1a, GR A),

## The EBCTCG meta-analysis 2011 [27]

In its recent quinquennial meta-analysis update, the Early Breast Cancer Trialists’ Collaborative Group (EBCTCG) included 17 randomized studies comparing postoperative radiotherapy vs. none and comprised 7 new studies in addition to previously reported trials. A total of 10,801 patients with pT1–2 tumors were recorded: the majority of whom (n = 7287) were node negative, 1050 were node positive, and in 2464 patients the nodal status was unknown.

The effect of radiotherapy on 10-year recurrences of any type and their relation to the 15-year breast cancer death rates were studied in correlation to various prognostic parameters and treatment characteristics. The absolute risk of any first recurrence was adjusted for trial, individual follow-up year, nodal status, and age (five groups), and also for tumor grade, tumor size, estrogen-receptor (ER) status, and whether or not tamoxifen had been used in both randomized groups.

Overall, WBI reduced the 10-year recurrence rate (local or distant) from 35 to 19.3 %, corresponding to an absolute benefit of 15.7 % (2p < 0.0001) for irradiated women (pN0: 15.4 %, pN+: 21.2 %). The 10-year rate of locoregional recurrence as the first event was substantially higher for non-irradiated women: 25.1 vs. 7.7 %, i.e., an absolute difference of 17.4 % (pN0: 15.5 %, pN+: 30.8 %). Moreover, WBI decreased the 15-year breast cancer death rate from 25.2 to 21.4 %, corresponding to an absolute gain of 3.8 % (pN0: 3.3 %, pN+: 8.5 %). Finally, radiotherapy reduced the 15-year risk of any death from 37.6 to 34.6 %, providing an absolute gain of 3.0 % (pN0: 2.8 %, pN+:10.7 %). Mortality without recurrence was slightly but not significantly higher in irradiated women [relative risk (RR) 1.09, 0.97–1.22, 2p = 0.14].

In summary, WBI halved the average annual rate of disease recurrence (RR 0.52, 0.48–0.56) and reduced the annual breast cancer death rate by about one sixth (RR 0.82, 0.75–0.90). On average, in all patients, about one breast cancer death was avoided per year 15 for every four recurrences avoided by year 10. Little variation of the proportional benefit was seen in the different prognostic subgroups. In contrast, the absolute benefit from radiotherapy substantially depended on the patient characteristics in terms of prognostic factors.

**Comments and conclusion of the DEGRO panel**

- This important large-scale meta-analysis impressively confirmed that prevention of locoregional recurrences by postoperative radiotherapy translates into improved survival.

## Radiotherapy in the elderly

The indication for WBI for women over 70 years with low risk tumors is an ongoing issue of international debate with partially antipodal interpretations [77]. The updated recommendations of the European Society of Breast Cancer Specialists (EUSOMA) [14] and the latest German S3 guidelines define no age limitation for postoperative RT for healthy elderly patients. Albeit the absolute benefit of adjuvant treatment is smaller in advanced age, the proportional risk reduction by half is constantly observed [3, 17, 22, 24, 27, 41, 66, 68, 69, 75, 84, 88, 89, 93, 94, 96]. In the EBCTCG 2011 analysis, even in node-negative patients over 70 years the 10-year recurrence rate was reduced from 17.7 to 8.8 % [27].

Data from population-based analyses reveal that refraining from RT in elderly patients is relatively common, consecutively leading to higher breast cancer mortality [38, 91].

No subgroup has yet been identified in whom patients did not profit from RT after BCS in terms of improved local tumor control. Therefore, omission of RT in patients of advanced age even with favorable prognostic factors (pN0, ER-positive, low grade) should only be considered in presence of comorbidities with a substantial reduction of life expectancy. This decision has to be properly documented [9, 35, 38, 75, 81, 85].

**Comments and conclusion of the DEGRO panel**

- Chronological age alone is not an appropriate criterion for decision against or in favor of adjuvant treatment.
- No subgroup of elderly patients has yet been identified that did not profit from RT in terms of local control.
- The DEGRO breast cancer expert panel explicitly discourages determination of a certain age for the omission of postoperative RT in healthy elderly women with low risk breast cancer.
- In frail elderly women omission of postoperative RT should be individually decided on the basis of geriatric assessment.

## Tumor bed boost

 

**Statement RT 2d** [74]

A local dose escalation (boost) to the tumor bed reduces local recurrence rates without demonstrating a survival advantage. A tumor bed boost is generally indicated (LoE 1a, GR A).

**Statement RT 2e** [74]

In postmenopausal patients with very low risk tumors (in particular > 60 years of age, small tumors, good prognostic features), the absolute benefit of a tumor bed boost is smaller. In this subgroup, its omission may be considered (LoE 2a, GR C),

##  

Considering the tumor bed as the area at highest risk for subclinical tumor cell contamination, a local dose escalation following WBI with 50 Gy has proven to decrease in-breast recurrence rates most effectively. The beneficial impact of an additional 16 Gy boost dose to the tumor bed by either fractionated external beam treatment or brachytherapy was corroborated in a follow-up analysis of the EORTC trial data, where local recurrence rates were consistently shown to be halved in every patients’ age group compared to WBI alone [4, 8, 67]. The highest benefit in terms of absolute risk reduction was demonstrated in younger women and patients at higher risk of local relapse. The boost technique (electrons, external beam photons or brachytherapy) had no significant impact on the oncologic outcome [44]. In this trial, higher local control rates achieved by boost radiotherapy did not translate into better survival.

Annual recurrence rates have decreased steadily during the last 10 years, due to quality progress in diagnostics, surgery, pathologic work up, frequent use of modern systemic therapy, and particularly, rapid innovation in radiotherapy. Interim reports of the ongoing prospective Young Boost Trial (NCT0021212) or closed ELIOT trial (WBI group) [62] describe low recurrence rates of estimated 0.5 % after 4 years (age group < 50 years) and 0.7 % after 5 years, respectively [7, 65], pointing at the value of further dose augmentation in the tumor bed.

The following techniques are used for boost treatment: external beam photons and electrons, interstitial and endoluminal brachytherapy, and intraoperative radiotherapy with electrons (IOERT) or in selected cases with low-energy photons (kV). Intraoperative, interstitial, and endoluminal techniques differ enormously in terms of dose distribution. Thus, outcome analyses of local control rates have to be performed according to the used method. IOERT has been demonstrated to be the method delivering the utmost uniform dose distribution within a given target volume [55]. Boost treatments by protons are considered to be experimental.

In the EORTC boost trial a dose of 16 Gy was used either in 8 fractions of 2 Gy EBRT or as low dose rate brachytherapy using 192-iridium implants with a dose rate of 0.5 Gy/h. In the French randomized trial, Romestaing et al. [71] applied 10 Gy in 2 Gy single doses; updated results have not been published. A subgroup analysis of the EORTC boost trial [67] assessed the outcome of 251 patients with R1 resection who were randomized to receive either a 10 Gy or a 26 Gy boost. There was no statistically significant difference in local control or survival between the lower and the higher boost dose group. Fibrosis occurred significantly more often after 26 Gy. Extrapolating these results on patients with complete resection, it seems justifiable to use a 10 Gy boost, even though data are less valid than for 16 Gy [74]. In a European survey about current practice in breast RT, one third of the institutions used a 10 Gy boost dose [90].

In recent years, an additional focus of boost therapy has been placed on accelerated dose integration during the course of WBI, which is possible using different approaches: during simultaneously integrated boosts (SIB, concomitant boost), the boost dose is administered by daily zonal dose augmentations [32, 78]. Intraoperative treatments precede WBI and are performed either with kV-photons or electrons. For IOERT, long-term follow-up data are available, indicating excellent high local tumor control rates in every risk group [29].

**DEGRO comments and conclusions**

- A boost in addition to WBI reduces local recurrence in all age groups and should, therefore, be offered to patients who appear biologically and mentally fit enough to experience the benefit of improved local control.
- For the remaining patients especially when they are > 60 years with small, node-negative, hormone receptor-positive tumors, omission of a boost may be considered.
- Regarding SIB techniques within normofractionated WBI, single tumor bed doses of 2.1 Gy for low-risk tumors (equal to a sequential boost with 5 × 2 Gy to 10 Gy) up to 2.25 Gy for constellations with higher risk of local recurrence (equal to a boost with 8 × 2 Gy to 16 Gy) seem to be within the acceptable range.

## Hypofractionated radiotherapy in breast cancer treatment

 

**Statement RT 2c** [74]

In elderly patients with tumors < 5 cm and without locoregional lymph node disease, who do not receive chemotherapy, as alternative to normofractionated WBI, hypofractionated schedules (e.g. 5 × 2.666 Gy/week up to 40 Gy) may be considered (LoE 1a, GR B),

##  

For breast cancer cells, a higher sensitivity towards single doses > 2 Gy has been postulated in radiobiological models, due to a lower alpha/beta (α/β) ratio with estimated values around 4 Gy in contrast to values of ten assumed for squamous cell carcinoma (SCC). This principle was exploited in whole breast radiotherapy (WBI) as well as accelerated partial breast irradiation (APBI) including intraoperative procedures (IORT) and SIB techniques [78].

Four randomized clinical trials (Start A, Start B, Owen, Whelan) investigated hypofractionated WBI schedules (39–42.9 Gy in single fractions of 2.6–3.3 Gy) for iso-effectiveness compared to normofractionation (50 Gy in single fractions of 2 Gy). In summary, 7095 patients were treated within these trials, 89.8 % with tumors < 3 cm, 79 % node negative, 87 % had small or medium-sized breasts. Only a minority was treated with chemotherapy, practically no experience exists with the use of taxanes in combination with HF-WBI.

In hypofractionated groups of the START trials, local recurrence rates at 5 and 10 years were reported in the range of 2.2–3.6 % and 9.1–14.8 %, respectively. In comparison to standard treatment arms, showing 5 and 10 year IBRs of 3–3.6 % and 12.1 %, respectively, corresponding results of the experimental arms showed a trend to be superior without statistical significance, providing equal long-term cosmetic outcome [11, 12, 63]. The 10-year follow-up data were presented at the SABCS 2012, corroborating the previous reports [39].The Canadian trial also confirms these findings in long-term analyses [95], with a possible exception of high-graded tumors, where hypofractionated schedules resulted in lower tumor control rates. This was in contrast to other data [40]. To date, ultrashort WBI courses are tested in the FAST trial (*fast*er radiotherapy for breast cancer), where a 5-fraction schedule of whole breast radiotherapy is compared against the UK standard 15-fraction regimen in terms of local cancer control and late adverse effects [2].

Cosmesis after 5 years was evaluated in about half of the patients. Long-term follow-up > 5 years was published in one study [40]. In a Cochrane meta-analysis [43], the authors stated that the findings of their review provided reassurance that offering HF to carefully selected patients is unlikely to be detrimental in terms of local control, breast appearance, survival, or late radiation breast toxicity for women with small to medium breasts, aged greater than 50 years, with small, node-negative tumors. The authors emphasize that longer follow-up is needed to finally assess the effects of HF. In accordance with these concerns, the ASTRO consensus and the German guidelines additionally recommend to refrain from HF when a homogenous dose is not achievable [74, 80].

Moreover, the impact of HF on late cardiac toxicity is not yet evaluated beyond 10 years [57, 95]; as the latency for clinical manifestation of cardiovascular effects is 15 years or longer, HF might turn out to be critical in cases of relevant dose exposure to the heart, especially in women with a longer life expectancy.

Conventional fractionation with a total dose of 50–50.4 Gy in single doses of 1.8–2 Gy is still regarded as standard in all current guidelines [9, 56, 74]. Divergence exists concerning the appraisal of hypofractionation as a routinely applicable alternative [9, 74] or as an option restricted to selected patients [74, 80].

Unresolved issues address the effectiveness in selective histologic subtypes (e.g., G3), a potentially worse cosmetic outcome in (very) large breasts and late normal tissue tolerance, especially in combination with modern chemotherapy regimen. Radiobiological models and first clinical results seem to predict inferior outcome for the ongoing ultrashort WBI trials [2].

**Comments and conclusion of the DEGRO panel**

- Normofractionated WBI plus sequential boost remains standard.
- Hypofractionated WBI with single doses up to 2.7 Gy in 15–16 fractions to total doses of 40–42 Gy is an option for older women with pT1–2 pN0 tumors who need no chemotherapy. The additional use of a sequential boost is possible.
- Hypofractionated WBI plus boost either by SIB or by hypofractionated sequential application is discouraged outside clinical trials.

## Targeting, treatment planning, and technique for the WBI

Individual CT-based 3D planning is mandatory. Several anatomically-based instruction guidelines have been published for definition of the planning target volume (PTV) [58, 73] which includes the mammary gland and the adjacent chest wall. Organs at risk (OAR) like lung and heart (in left-sided tumors possibly with delineation of the left anterior coronary artery) [30, 58] are contoured and doses documented in dose–volume histograms (DVH). For planning and treatment, patients usually are placed in supine position with elevated arms in immobilization cradles.

For WBI, homogeneity requirements in dose distribution as described by the ICRU reports 50 and 62 are usually achieved by conformally shaped tangential wedged field techniques, mostly with photons at energies of 4–8 MV. For larger breasts, higher energies and/or intensity modulation (IMRT) within the tangential beams may be appropriate to fulfill the minimum homogeneity criteria [6, 42]. Multi-angle IMRT may be beneficial for patients with difficult anatomical conditions (i.e., funnel chest) where tangential field arrangements would lead to excessive exposure of OARs. However, the routine use of multi-angle IMRT techniques is problematic due to the increased volume exposed to low doses [50]. Therefore, a potentially increased risk of secondary tumor induction must be weighed against sparing of OAR [26, 37]. In selected cases, WBI in prone position might also serve as alternative [45]. The use of active breathing control, such as computer-assisted breath hold in deep inspiration [83, 99] provides another possibility of sparing radiation burden to the lungs and for left-sided tumors also to cardiac structures as it increases the distance between target volume and heart [49].

For quality control, correct treatment delivery has to be documented with repeated imaging, mostly by EPIDs or kV-based image guidance, alternatively by optical surface scanning techniques [34].

## Radiotherapy following neoadjuvant chemotherapy and breast conserving surgery

 

**Statement RT 4d** [74]

After primary (neoadjuvant) systemic therapy, the indication for adjuvant radiotherapy follows pretherapeutic TN-categories irrespective of response to the primary systemic treatment (LoE 2A, GR A).

##  

The abovementioned statement is part of the S3 recommendations concerning postmastectomy radiotherapy (PMRT). Of note, the S3 guidelines do not explicitly provide information on adjuvant radiotherapy after breast conserving surgery subsequent to primary systemic treatment (PST). Usually, patients referred to PST are also considered to be at higher risk for a locoregional relapse, thus, emphasizing the need for an amplified locoregional treatment. Hence, and in the absence of randomized prospective trials, indications for radiotherapy following BCS are not altered by the time sequencing of the systemic medications.

A major goal of neoadjuvant chemotherapy in advanced breast cancer cases is to facilitate breast conserving treatment without compromising local control (LC) and survival rates. Prospective and retrospective data of the 1990s up to 2010 verified the feasibility of a multimodality treatment including WBI after breast conserving surgery as an indispensable component. However, its optimal use is a matter of controversy due to presumably higher regional recurrence (RR) and in-breast relapse (IBR) rates which were reported in a dimension of 10.5 % (range 1.8–22.5) and 8 % (range 1.8–17), respectively, after median follow-up periods of 5 years (range 29–124 months) [13, 15, 19, 20, 21, 31, 51, 52, 53]. Omission of surgery resulted in higher LR rates [51].

During the last decade, data on lowest locoregional recurrence (LRR) rates following postoperative radiotherapy after neoadjuvant CT were provided by two retrospective studies [13, 20, 21]. WBI was performed in conventional fractionation (1.8–2 Gy) up to a total dose of 45–50 Gy, followed by a fractionated tumor bed boost with external electrons with doses between 10 and 20 Gy. In these trials, pathological complete response (pCR) rates around 23.5 % were achieved after PST. At median follow-up times of 60 months, compiled rates (mean values) of LRR, IBR, and distant metastasis amounted to 8, 4.5, and 19 %, respectively. In contrast, results of prospective trials are less favorable, reporting mean IBR rates of about 9.6 % after a median follow-up of 68.4 months (range 29–124 months) [15, 18, 19, 31, 52, 72]. However, these reports lack precise information on radiotherapy details (e.g., boost techniques and their dose prescription). Improvement of local control by radiation dose escalation (e.g., by IOERT) is a promising strategy, but is associated with possible impairment of cosmesis [28].

**Conclusion of the DEGRO panel**

- Normofractionated WBI following PST (primary systemic therapy) is mandatory during BCT irrespective of achieved response.
- A focus of interest is the investigation of dose augmentation to the tumor bed (e.g., by altered fractionation and/or total dose escalation) for possible compensation of higher local relapse rates.

## Partial breast irradiation

 

**Statement RT 3** [74]

Radiotherapy restricted to parts of the affected breast (partial breast irradiation, PBI) as sole radiation treatment including sole intraoperative radiotherapy (IORT) represents no treatment standard (LoE 3b).

##  

Accelerated partial breast irradiation (APBI) refers to RT of a smaller (partial) breast volume over a shorter time interval, covering the tumor bed with a limited margin of normal tissue. For patients at a lower risk for local recurrence, APBI has been strongly propagated in recent years as it spares treatment time, costs, and allegedly provides less toxicity [54]. APBI can be delivered intra-operatively in a single fraction or postoperatively over 1–3 weeks by brachytherapy or external beam radiotherapy. In analogy to boost settings, these methods differ in terms of physical dose distribution and radiobiologic effects, which has to be considered in outcome analyses [55, 59].

## Cinical evidence

So far, only four phase III randomized trials and one meta-analysis evaluating APBI have been published. Three of these studies have important limitations: The Yorkshire Breast Cancer Group trial [25] failed to complete accrual and used a variety of techniques. In the Christie Hospital trial [70], 708 patients were randomized between APBI and WBI, but lacked appropriate target volume definition criteria; moreover, many patients outside the low-risk group were entered. The Hungarian trial comparing WBI with APBI using HDR implants or electrons (normal fractionation) was prematurely stopped after recruitment of 258 women with margin-negative, early-stage breast cancers, thus, lacking sufficient sample size [64]. A meta-analysis of these phase III trials was published in 2010 [87] Compared to WBI, APBI was associated with an increased risk for both local [pooled odds ratio (OR) 2.15; p = 0.001] and regional recurrence (pooled OR 3.43; p < 0.001), however, not (yet) translating into a survival difference (OR 0.91; p = 0.55) [54]. Orthovoltage IORT as APBI strategy was investigated in the TARGIT trial as the fourth published randomized investigation and is discussed below.

### Intraoperative radiotherapy

To date, two major “full-dose” IORT studies are promoted: the TARGIT approach using ortovoltage x-rays and the ELIOT trial, respectively, testing single-shot electron treatment. In 2010, a first interim analysis was published from the TARGIT trial at a median follow-up time of 24.6 months [86].The Kaplan–Meier estimate of local recurrence at 4 years was 1.2 % in the APBI arm and 0.95 % in the WBI group. The latest update was presented at the 2012 SABCS, with a publication awaited. In this analysis of 3,442 patients and a median follow-up of 29 months, the local failure rate in the TARGIT group was 2 % higher in comparison to WBI (p = 0.042, HR = 2.05). The frequency of any complications and major toxicity was similar with both treatment modalities (3.3 % TARGIT vs. 3.9 % WBI).

The ELIOT trial [61] has reached its accrual goal, 651 ELIOT patients were randomly compared to a cohort treated with standard WBI, with a publication pending. First results have been presented, alerting a higher IBTR rate in the IORT arm [62].Outside this trial setting, another 1822 patients were treated using the ELIOT concept [92]. After a median follow-up of 36 months, altogether 3.63 % in-breast recurrences were observed. Predictive factors for LR were age < 50 years, tumor size, grading, involved nodes and negative hormone receptors. When analyzed according to current ASTRO Consensus Statement Guidelines for the application of accelerated partial breast irradiation, the 5-year rate of ipsilateral breast recurrence for suitable, cautionary, and unsuitable groups were 1.5, 4.4, and 8.8 %, respectively (p = 0.0003) [48]. Applying GEC-ESTRO risk criteria, the 5-year rate of in-breast tumor reappearances for “good candidates” amounted to 1.9 %, for “possible candidates” and “contraindication” 7.4 and 7.7 %, respectively (p 0.001) [47].

In light of the existing literature, it seems premature to interpret the results following sole IORT—by any means—as isoeffective compared to standard treatment. True local recurrences are presumed to occur between 40 and 65 months after primary treatment [16, 36], out-quadrant relapses even later than that [33] when WBI was performed. Only adequate long-term experience will reveal the potential of a sole IORT approach to replace WBI in selected patient groups [76].

## Summary

To date, the results of seven randomized phase III trials are awaited [5, 10, 61, 62, 82, 97, 98], three of them having completed accrual [10, 62, 98] A direct clinical comparison of APBI techniques would be highly desirable, but has not yet been undertaken by any of the mentioned trials. Results with follow-up over 5 years will not be available until 2017, hopefully not only providing information regarding the long-term efficacy and safety of APBI, but also in terms of adequate patient selection and dose–volume constraints [54]. Nonetheless, ASTRO as well as GEC-ESTRO permit APBI for selected patients [64, 79]. The ASTRO Task Force “strongly endorsed enrollment of all eligible patients considering APBI into prospective clinical trials to address many of the unanswered questions in APBI” [79]. The German S3 guidelines still do not recommend APBI outside clinical studies [1, 74].

**Comments and conclusions of the DEGRO panel**

- Optimal selection criteria for sole APBI as a personalized RT approach are still not well defined. In addition to conventional clinical tumor characteristics, molecular and genetic profiles have to be investigated for their role in the patient selection process.
- To date, clinical studies reveal higher local recurrences rates after APBI in comparison to WBI in all risk settings.
- Patients should be carefully selected for APBI within prospective trials, also addressing newer prognostic markers.
- Justification of APBI alone for selected patients beyond clinical trials was assessed controversially among the panel members. The majority of the panel finally agreed to concede APBI with established techniques like multicatheter brachytherapy or IORT as an option for elderly women fulfilling all of the following preconditions: age > 70 years, tumor size < 2 cm, invasive ductal carcinoma, negative axillary nodes, free surgical margins, absence of EIC and luminal A type (ER+ and PR+, G1–2, Her2neu negative). The patient has to be informed about a modest reduction of in-breast tumor control rates. Meticulous follow-up and documentation of outcome in the framework of a certified breast cancer center are mandatory.

## References

[1] AGO (2013) Diagnostik und Therapie primärer und metastasierter Mammakarzinome. Adjuvante Strahlentherapie. (Version 2013.1.D). http://www.ago-online.de. Arbeitsgemeinschaft Gynäkologische Onkologie

[2] Agrawal (2011). Radiother Oncol.

[3] Anderson (2009). J Clin Oncol.

[4] Antonini (2007). Radiother Oncol.

[5] Armaroli L, Barbieri E, Bertoni F et al (2007) Breast cancer with low risk of local recurrence: partial and accelerated radiation with three-dimensional conformal radiotherapy (3DCRT) vs. standard radiotherapy after conserving surgery (phase III study). Version 01.05.2007. http://groups.eortc.be/radio/res/irma/synopsis_trial_irma1.pdf. Accessed 27 August 2013

[6] Barnett (2012). Int J Radiat Oncol Biol Phys.

[7] Bartelink (2012). Radiother Oncol.

[8] Bartelink (2007). J Clin Oncol.

[9] Belkacémi (2011). Crit Rev Oncol Hematol.

[10] Belkacemi Y, Lartigau E, Bourgier C (2013) Standard of hypofractionated radiotherapy versus accelerated partial breast irradiation (APBI) for breast cancer (SHARE). http://www.clinicaltrials.gov/ct2/show/NCT01247233. Accessed 27 Aug 2013

[11] Bentzen (2008). Lancet.

[12] Bentzen (2008). Lancet Oncol.

[13] Beriwal (2006). Breast J.

[14] Biganzoli (2012). Lancet Oncol.

[15] Bonadonna (1998). J Clin Oncol.

[16] Brooks (2005). Int J Radiat Oncol Biol Phys.

[17] Buchholz (2006). J Clin Oncol.

[18] Calais (1994). Cancer.

[19] Cance (2002). Ann Surg.

[20] Chen (2004). J Clin Oncol.

[21] Chen (2005). Cancer.

[22] Clarke (2006). Ann Oncol.

[23] Clarke (2005). Lancet.

[24] Darby (2011). Lancet.

[25] Dodwell (2005). Clin Oncol.

[26] Donovan (2012). Med Phys.

[27] Early (2011). Lancet.

[28] Fastner (2013). Strahlenther Onkol.

[29] Fastner G, Sedlmayer F, Merz F et al (2013) IORT with electrons as boost strategy during breast conserving therapy in limited stage breast cancer: long term results of an ISIORT pooled analysis. Radiother Oncol. doi:pii:S0167-8140(13)00272-7. 10.1016/j.radonc.2013.05.031 (Epub ahead of print) 10.1016/j.radonc.2013.05.031 23830467

[30] Feng (2011). Int J Radiat Oncol Biol Phys.

[31] Fisher (1998). J Clin Oncol.

[32] Freedman (2007). Int J Radiat Oncol Biol Phys.

[33] Freedman (2005). Int J Radiat Oncol Biol Phys.

[34] Gaisberger C, Steininger P, Mitterlechner B et al (2013) Three-dimensional surface scanning for accurate patient positioning and monitoring during breast cancer radiotherapy. Strahlenther Onkol (Epub ahead of print) 10.1007/s00066-013-0358-6 23740155

[35] Giordano (2005). J Clin Oncol.

[36] Gujral (2011). Int J Radiat Oncol Biol Phys.

[37] Hall (2003). Int J Radiat Oncol Biol Phys.

[38] Hancke (2010). Ann Oncol.

[39] Haviland JS, Agrawal R, Aird E et al (2012) The UK START (Standardisation of Breast Radiotherapy) Trials: 10-Year Follow-Up Results. SABCS S4-1

[40] Herbert (2012). Int J Radiat Oncol Biol Phys.

[41] Hughes (2013). J Clin Oncol.

[42] ICRU Report 62. Prescribing, recording and reporting photon beam therapy (Supplement to ICRU Report 50). International Commission on Radiation Units and Measurements, 1999

[43] James (2010). Cochrane Database Syst Rev.

[44] Jones (2009). J Clin Oncol.

[45] Kirby (2011). Radiother Oncol.

[46] Leitlinienprogramm Onkologie DKG, Deutsche Krebshilfe, AWMF (2012) Interdisziplinäre S3-Leitlinie für die Diagnostik, Therapie und Nachsorge des Mammakarzinoms. http://www.senologie.org/fileadmin/downloads/S3-Brustkrebs-v2012-OL-Langversion.pdf. Accessed 27 Aug 2013

[47] Leonardi (2013). Radiother Oncol.

[48] Leonardi (2012). Int J Radiat Oncol Biol Phys.

[49] Lin (2008). Int J Radiat Oncol Biol Phys.

[50] Lohr (2009). Int J Radiat Oncol Biol Phys.

[51] Mauri (2005). J Natl Cancer Inst.

[52] Mauriac (1999). Ann Oncol.

[53] Min (2011). Int J Radiat Oncol Biol Phys.

[54] Moser (2012). Breast.

[55] Nairz (2006). Strahlenther Onkol.

[56] National Comprehensive Cancer Network (2013) NCCN Clinical Practice Guidelines in Oncology: Breast Cancer, Version 1.2013. http://www.nccn.org/professionals/physician_gls/f_guidelines.asp#site. Accessed 27 Aug 2013

[57] NICE (2009). National Institute for Clinical.

[58] Nielsen (2013). Acta Oncol.

[59] Njeh (2010). Radiat Oncol.

[60] Ontario Clinical Oncology Group, Canadian Institutes of Health Research, Canadian Breast Cancer Research Alliance. RAPID: randomized trial of accelerated partial breast irradiation. http://www.clinicaltrials.gov/ct2/show/NCT00282035. Accessed 27 August 2013

[61] Orecchia (2003). Breast.

[62] Orecchia (2012). Radiother Oncol.

[63] Owen (2006). Lancet Oncol.

[64] Polgár (2007). Int J Radiat Oncol Biol Phys.

[65] Poortmans (2012). Semin Radiat Oncol.

[66] Poortmans (1). Radiother Oncol 2007.

[67] Poortmans (2009). Radiother Oncol 2009.

[68] Pötter (2007). Int J Radiat Oncol Biol Phys.

[69] Prescott (2007). Health Technol Assess.

[70] Ribeiro (1993). Clin Oncol.

[71] Romestaing (1997). J Clin Oncol.

[72] Rouzier (2001). J Clin Oncol.

[73] RTOG (2013) A Breast cancer atlas for radiotherapy planning. Consensus definitions http://www.RTOG.org. Accessed 27 Aug 2013

[74] S3 (2012) Interdisziplinäre S3-Leitlinie für die Diagnostik, Therapie und Nachsorge des Mammakarzinoms, Langversion 3.0, Aktualisierung 2012, AWMF-Register-Nummer: 032 – 045OL

[75] Sautter-Bihl (2011). Dtsch Arztebl Int.

[76] Sautter-Bihl (2010). Strahlenther Onkol.

[77] Sautter-Bihl (2012). Strahlenther Onkol.

[78] Sedlmayer (2013). Strahlenther Onkol.

[79] Smith (2009). Int J Radiat Oncol Biol Phys.

[80] Smith (2011). Int J Radiat Oncol Biol Phys.

[81] Smith (2013). J Clin Oncol.

[82] Strnad V, Polgár C, on behalf of the European Brachytherapy Breast Cancer GEC-ESTRO Working Group (2013) Phase III multicenter trial. Interstitial brachytherapy alone versus external beam radiation therapy after breast conserving surgery for low risk invasive carcinoma and low risk duct carcinoma in-situ (DCIS) of the female breast. http://www.apbi.uni-erlangen.de. Accessed 27 August 2013

[83] Swanson (2013). Am J Clin Oncol.

[84] Tinterri (2009). Breast.

[85] Truong (2006). Am J Surg.

[86] Vaidya (2010). Lancet.

[87] Valachis (2010). Breast J.

[88] Van (2004). Radiother Oncol.

[89] Van (2000). Radiother Oncol.

[90] Van (2010). Radiother Oncol.

[91] Varga D, Wischnewsky M, Atassi Z, et al (2010) Does guideline-adherent therapy improve the outcome for early-onset breast cancer patients? Oncology 78(3–4):189–195 10.1159/000313698 20414007

[92] Veronesi (2010). Breast Cancer Res Treat.

[93] Vinh-Hung (2004). J Natl Cancer Inst.

[94] Weaver (2011). N Engl J Med.

[95] Whelan (2010). N Engl J Med.

[96] Wildiers (2007). Lancet Oncol.

[97] Wolmark N, Curran WJ, Vicini F et al (2013) NSABP protocol B-39. RTOG protocol 0413. A randomized phase III study of conventional whole breast irradiation (WBI) versus partial breast irradiation (PBI) for women with stage 0, I, or II breast cancer. http://www.rtog.org/members/protocols/0413. Accessed 27 Aug 2013 17111558

[98] Yarnold J, Coles C, on behalf of the IMPORT LOW Trial Management Group (2013) Intensity modulated and partial organ radiotherapy. Randomised trial testing intensity modulated and partial organ radiotherapy following breast conservation surgery for early breast cancer. http://www.clinicaltrials. gov/ct2/show/NCT00814567. Accessed 27 Aug 2013

[99] Zurl (2010). Strahlenther Onkol.

